# Charles Bonnet syndrome: predisposing factors and more effective pharmacology therapy

**DOI:** 10.1192/j.eurpsy.2025.667

**Published:** 2025-08-26

**Authors:** E. I. Koiliari, I. Mouzas, E. L. Pasparakis

**Affiliations:** 1 Department of Psychiatry, General Hospital of Agios Nikolaos of Lasithi, Agios Nikolaos of Lasithi; 2 Department of Pathology, Laboratory of Alcohology of the Medical School of the University of Crete; 3 Department of Pathology, University General Hospital of Irakleion (PAGNI), Herakleion, Greece

## Abstract

**Introduction:**

Charles Bonnet syndrome (CBS) is a condition in which patients with a free psychiatric history manifest visual hallucinations on the basis of a diagnosed ophthalmic disease (brain disease or optic nerve damage), which causes severe vision loss (partial or total).

**Objectives:**

Hypothesis testing: “Administration of low doses of typical and atypical antipsychotics is the more effective pharmacological treatment for visual hallucinations of CBS”.

**Methods:**

Patients underwent pathological, psychiatric and ophthalmological assessment, resulting in a complete study-appropriate medical history.

**Results:**

The sample consisted of ten female patients and eight male patients. The median age of the sample was 84 years. None of the patients was diagnosed with major psychopathology and in no clinical case was an individual medical history of dependence on alcohol or other psychotropic substances recorded. A common cause of the dysfunctional vision of the entire sample of patients was the visual deficit due to a diagnosed cataract condition. During the examination of each patient, it was found that a significant number of predisposing factors for CBS, as described in the literature, were detected. Increased stress levels, depressed mood, cognitive deficits, damage at the level of brain tissue, living conditions of the patient in environments with insufficient light intensity, social withdrawal or poor interaction with others contributed to the vulnerability of visual hallucinations. The administration of broad-spectrum medicines of Internal Medicine was also recorded as a covariate for the triggering of visual hallucinations. As demonstrated by the majority of patients, the administration of a low dose of a typical antipsychotic led to effective treatment of the syndrome.

**Conclusions:**

Based on the present study, in patients of both sexes (men and women), aged 73-98 years, haloperidol and risperidone in low doses of 1-2 mg and olanzapine in low doses of 2.5-5 mg appear to be more effective options in treating with optical hallucinations in CBS.

**Disclosure of Interest:**

None Declared

